# Information theoretic approach to complex biological network reconstruction: application to cytokine release in RAW 264.7 macrophages

**DOI:** 10.1186/1752-0509-8-77

**Published:** 2014-06-25

**Authors:** Farzaneh Farhangmehr, Mano Ram Maurya, Daniel M Tartakovsky, Shankar Subramaniam

**Affiliations:** 1 Department of Bioengineering, University of California San Diego, 9500 Gilman Drive, 92093-0412 La Jolla, CA, USA; 2 Department of Mechanical and Aerospace Engineering, University of California San Diego, 9500 Gilman Drive, 92093-0411 La Jolla, CA, USA; 3 San Diego Supercomputer Center, University of California San Diego, 9500 Gilman Drive, 92093-0505 La Jolla, CA, USA; 4 Departments of Chemistry & Biochemistry, Cellular and Molecular Medicine and Graduate Program in Bioinformatics, University of California, San Diego, 9500 Gilman Drive, La Jolla, CA, USA

**Keywords:** Bioinformatics, Data mining, Network inference, Data-driven network reconstruction, Information theory, Mutual information, Probabilistic algorithm, Statistical methods

## Abstract

**Background:**

High-throughput methods for biological measurements generate vast amounts of quantitative data, which necessitate the development of advanced approaches to data analysis to help understand the underlying mechanisms and networks. Reconstruction of biological networks from measured data of different components is a significant challenge in systems biology.

**Results:**

We use an information theoretic approach to reconstruct phosphoprotein-cytokine networks in RAW 264.7 macrophage cells. Cytokines are secreted upon activation of a wide range of regulatory signals transduced by the phosphoprotein network. Identifying these components can help identify regulatory modules responsible for the inflammatory phenotype. The information theoretic approach is based on estimation of mutual information of interactions by using kernel density estimators. Mutual information provides a measure of statistical dependencies between interacting components. Using the topology of the network derived, we develop a data-driven parsimonious input–output model of the phosphoprotein-cytokine network.

**Conclusions:**

We demonstrate the applicability of our information theoretic approach to reconstruction of biological networks. For the phosphoprotein-cytokine network, this approach not only captures most of the known signaling components involved in cytokine release but also predicts new signaling components involved in the release of cytokines. The results of this study are important for gaining a clear understanding of macrophage activation during the inflammation process.

## Background

Cellular functions and biological processes are regulated by complex biochemical reactions within and between the cells [1,2]. Bimolecular techniques can be used to measure concentrations of various molecular components, such as proteins and metabolites, allowing a partial reconstruction of the networks involving these components. A goal of systems biology is to reconstruct these underlying networks and to infer associated biological phenomena from large scale measurements [3]. More specifically, reconstruction of biological networks yields a framework for understanding the relationship between molecular measurements and higher-level phenotypes [4,5].

Analyses of diverse read-outs from cells allow one to map an input onto responses associated with a given phenotype, i.e., to reconstruct the underlying biological network that results in the phenotype. Current computational approaches for network reconstruction include principal component regression (PCR) [6], partial least squares (PLS) regression [7], linear matrix inequalities (LMI) [8], and Bayesian Networks (BNs) [9]. These approaches are briefly described below.

PCR is a regression procedure that uses a principal component analysis to estimate regression coefficients [6]. Usually, principal components with the highest variance are selected in three steps. First, a principal component analysis is performed on the data matrix of explanatory variables. Second, a least-squares regression is applied between the selected components (latent variables) and the output/response variables. Finally, the model’s parameters are calculated for the selected explanatory variables by combining the two steps [10]. In contrast to PCR, PLS regression captures the maximum variance in the output variables while capturing sufficient variance in the input variables [7,11]. PLS makes a linear model by projecting the input and output variables onto a new space [12,13]. LMI converts nonlinear convex optimization problems into linear optimization problems [14]. The basic idea of the LMI is to approximate a given input/output modeling problem posed as a quadratic optimization problem with a linear objective and so-called LMI constraint [8]. Approaches such as PCR and PLS essentially work based on a linear model template. Bayesian networks are graphical models that describe causal or pseudo-causal interactions between variables [9,15]. Nodes of a BN represent random variables in the Bayesian sense and edges represent conditional dependencies among the random variables [16]. BNs have a number of drawbacks related to the so-called representation problem: they require one to choose between discrete or continuous variables and parametric or non-parametric forms of the conditional probability distribution, and to decompose the joint probability distribution into conditional probability distributions among the relevant variables [17]. Information-theoretic approaches provide a non-parametric alternative to Bayesian networks. They construct parsimonious models of biological networks by establishing statistical dependencies of interactions based on their uncertainty reductions [18-20]. Unlike PCR/PLS, this approach does not make any assumptions about the linearity of the system and the functional form of the statistical distribution of the variables [21,22]. We describe our information-theoretic approach to the reconstruction of biological networks in the next section. Next, this method is used to develop a parsimonious model of phosphoprotein-cytokine network in RAW 264.7 macrophages. In the following sections, we compare the regulatory components captured by our approach with those identified by previous approaches and the knowledge available in scientific literature.

### Shannon’s information theory

Building upon Hartley’s conceptual framework [23], which relates the information of a random variable with its probability, Shannon [24] defined “entropy” of a random variable in terms of its probability distribution. For a random variable *X* given a random sample {*x*_1_, …, *x*_*n*_} with probabilities *P*(*x*_*i*_), entropy H is defined as

(1) $$H\left(X\right)=-\sum_{i=1}^{n}P(x_{i})\log\left[P\left(x_{i}\right)\right]$$

Shannon’s information theory defines “mutual information” as the amount of information about a random variable *X* that can be obtained by observing another random variable *Y*. This definition implies that the information that *Y* provides about *X* reduces uncertainty about *X* due to the knowledge of *Y*. Intuitively, mutual information infers the information that *Y* and *X* share by measuring how much knowing one of the variables can reduce the uncertainty about the other [25]. Then, the mutual information of *Y* relative to *X*, or *X* relative to *Y*, is given by

(2) $$I\left(X,Y\right)=H\left(X\right)+H\left(Y\right)-H\left(X,Y\right)=I\left(Y,X\right)$$

Mutual information provides a metric for measuring statistical dependencies of interactions. It has several advantages over other methods [18-20]: It does not make any assumption about the functional form of the statistical distribution of variables [22]; and, information theoretic approaches are not dependent on the linearity assumption of the model for the ease of computation [21].

### Threshold selection on mutual information

A parsimonious model of a complex system has to capture a necessary and sufficient model of the entire system, while minimizing the number of interacting components, from the measured data for the system. The ultimate goal of data-driven network-reconstruction methods is to find such a necessary and sufficient model. Information theoretic approaches analyze the statistical dependencies of interacting components by measuring the mutual information coefficients of interactions. A mutual information network of a complex system is obtained by computing the mutual information matrix (MIM) and selecting the threshold of mutual information (TMI). MIM is a square matrix, whose elements *MIM*_*ij*_ = *I (X*_*i*_*, Y*_*j*_*)* are the mutual information between the variables *X*_*i*_ and *Y*_*j*_. TMI defines the threshold of statistical dependencies of interactions. Choosing an appropriate TMI is a non-trivial problem. A straightforward but computationally demanding approach is to perform permutations of measurements several times and to recalculate a distribution of the mutual information for each permutation. Then permuted distributions are averaged and the largest mutual information in the averaged permuted distribution represents the threshold [26]. Some of the algorithms for network reconstruction and threshold selection in biological networks are discussed below.

The Relevance Network (RelNet) constructs a network in which a pair of random variables *X*_*i*_ and *Y*_*j*_ is linked by an edge if the mutual information *I*(*X*_*i*_,*Y*_*j*_) is larger than a given threshold [27]. The Context Likelihood of Relatedness (CLR) algorithm derives a score from the empirical distribution of the mutual information for each pair of random variables *X*_*i*_ and *Y*_*j*_[28]. CLR estimates a score

$$W_{\mathrm{ij}}=\sqrt{z_{i}^{2}+z_{j}^{2}},z_{i}=\text{max}\left\{0,,,\;,\left[I,\;,\left(X_{i},X_{j}\right),-,{µ}_{i}\right],/,\sigma_{i}\right\}$$

where *μ*_*i*_ and *σ*_*i*_ are the mean and standard deviation of the distribution of the mutual information of *X*_*i*_ and all other variables *Y*_*j*_ (*j = 1,…,n*). The Minimum Redundancy Network (MRNet) relies on the conditional mutual information to make inference. MRNet is applied to determine regulatory targets and pathways. If two random variables *X* and *Y* have a large mutual information but are conditionally independent given a third random variable *Z*, MRNet considers no statistical dependency between them [29]. ARACNE (Algorithm for the Reconstruction of Accurate Cellular NEtworks) assigns to each pair of nodes a weight equal to their mutual information and removes the weakest edges by applying a proper threshold [30]. ARACNE applies Kernel Density Estimation (KDE) approaches to measure mutual information between nodes and selects the bandwidth of kernels by minimizing the Kullback–Leibler distances between kernel density distributions of variables before and after removing the *i*^th^ observation. It also applies an information-theoretic property called the Data Processing Inequality (DPI) to remove statistically weak connections. DPI states that, if *X*_*i*_ interacts with *X*_*j*_ through a random variable *X*_*k*_ then *I*(*X*_*i*_, *X*_*j*_) < *min*{*I*(*X*_*i*_, *X*_*k*_), *I*(*X*_*j*_, *X*_*k*_)}.

We employ an information-theoretic approach both to reconstruct complex biological networks and to establish a parsimonious model of the entire system. Our strategy is to determine mutual information of interactions using kernel density estimators based on an unbiased cross-validation [31-33] estimation of kernel bandwidths and to analyze statistical dependencies of nodes by selecting a threshold obtained by applying the large deviation theory [18] employed by ARACNE [30].

## Methods

### Information theoretic approach for biological network reconstruction

As mentioned before, MI measures the information that *X* and *Y* share by measuring how much knowing one of these variables will reduce the uncertainty of the other and reflects the statistical dependencies of two variables. Hence, higher MI between an input and an output indicates a larger reduction in uncertainty and suggests a stronger input–output connection. Small (statistically zero) MI between two random variables indicates that variables are independent.

Measuring mutual information with a kernel density estimator (KDE)—a non-parametric method for estimating probability densities of variables—is more advantageous than histogram-based methods in terms of a better mean square error rate of convergence of the estimate to the underlying density [32]. We note than ARACNE also uses KDE [30] to estimate MI. A disadvantage of KDE is the need to specify an optimal kernel bandwidth [33]. Once the optimal kernel bandwidth is obtained and the MI coefficients of the network are measured using KDE, the next step is to select a proper threshold to determine the boundary of statistically significant connections and the weak connections to be removed; similar concept of statistical significance has been used by Pradervand et al. [34] in a PCR-based approach to network reconstruction. Following these three steps, information theoretic model of the network is obtained. It provides a parsimonious network in which the number of false connections are reduced considerably. Our method of MI-based network reconstruction is inspired by (and borrows several components from) the ARACNE framework [30]. However, we employ a different methodology for the selection of optimal kernel bandwidth as described below.

The following subsections present a description of the above-mentioned steps to create a data-driven model of complex networks. These steps are applied to decipher, in a lumped manner, regulatory mechanisms involved in the release of seven cytokines by activation of 22 signaling proteins in RAW 264.7 macrophage. The Alliance for Cellular Signaling (AfCS) has generated a systematic profiling of signaling responses and cytokine release in RAW 264.7 macrophage [35,36]. This dataset consists of data from stimulation of macrophages by both Toll and non-Toll receptor ligands. The objective is to create an input–output model, in which signaling responses (22 inputs) are used to predict cytokine release (7 outputs).

### Non-parametric estimations of mutual information

Kernel Density Estimation (KDE) is a non-parametric method to determine the Probability Density Function (PDF) of a random variable. Given a random sample {*x*_1_, …, *x*_*n*_} for a univariate random variable *X* with an unknown density *f*, a kernel density estimator (KDE) estimates the shape of this function as [32]:

(3) $$f\left(x\right)=\frac{1}{\sqrt{2\pi }\mathrm{nh}}\sum_{i=1}^{n}\exp\left[-\frac{{\left(x-x_{i}\right)}^{2}}{2h^{2}}\right]$$

where, *h* is the kernel bandwidth. A bivariate kernel density function of two random variables *X* and *Y* given two random samples {*x*_1_, …, *x*_*n*_} and {*y*_1_, …, *y*_*n*_} is defined as:

(4) $$f\left(x,y\right)=\frac{1}{2\mathrm{\pi n}h^{2}}\sum_{i=1}^{n}\exp\left[-\frac{{\left(x-x_{i}\right)}^{2}+{\left(y-y_{i}\right)}^{2}}{2h^{2}}\right]$$

The mutual information of *X* and *Y* is computed as [37]:

(5) $$I\left(X,Y\right)=\frac{1}{n}\sum_{j=1}^{n}\ln\frac{f\left(x_{j},y_{j}\right)}{f\left(x_{j}\right)f\left(y_{j}\right)}$$

where, *n* is the sample size, and *h* is the kernel width.

### Selection of optimal kernel bandwidth

The use of KDEs to evaluate the MI coefficients requires the optimal selection of the kernel bandwidth *h*. The main criterion used to determine the optimal kernel width is the minimization of the expected risk function, defined as the mean integrated squared error (MISE) between the computed and true (unknown) distributions [32,33],

(6) $$\mathrm{MISE}\left(h\right)=E\int {\left[f_{h}\left(x\right)-f\left(x\right)\right]}^{2}\mathrm{dx}$$

where, *f*_*h*_*(x)* is the kernel density estimate of *x* for a bandwidth of *h*. MISE cannot be used directly since it involves the unknown density function *f(x)*. To address this issue, several algorithms have been developed to get an estimate of the optimal bandwidth. One of the most commonly used algorithms employs a cross-validation type approach. Based on this approach, if *f*_*h*_*(x)* is the kernel density estimation at *x* for a bandwidth of *h* using all of the data to fit the KDE, then a cross-validated estimate of the bandwidth is the value for *h* that minimizes [31-33]:

(7) $$\int f_{h}^{2}\left(x\right)\mathrm{dx}-\frac{2}{n}\sum_{i=1}^{n}f_{\left(-i\right),h}\left(x_{i}\right)$$

where, *f*_*(−i),h*_*(x*_*i*_*)* is the kernel density estimator using the bandwidth *h* at *x*_*i*_ obtained after removing *i*^th^ observation. For two vectors *X* and *Y*, the cross-validation method determines the optimal kernel width for each pair of randomly selected set of *n* pairs of variables and the mean of optimal kernel widths for these *n* pairs is used as an approximated kernel width for the entire dataset [38].

### Network reconstruction and threshold selection

Once the optimal kernel width has been selected and the MI matrix has been computed, the next step is to find an appropriate threshold of MI, *I*_*0*_. Based on large deviation theory used by ARACNE algorithm [30], the probability that an empirical value of mutual information *I* is greater than *I*_*0*_*,* provided that its true value is $\bar{I}=0$, is

(8) $$P\left(I>I_{0}\left|\bar{I}\right)=0\right)\sim e^{-\mathrm{cN}I_{0}}$$

where, *c* is a constant. Taking the natural logarithm of both sides yields

(9) $$\ln P=a+bI_{0}$$

where, *b* is proportional to the sample size *N*. Therefore, ln*P* is a linear function of *I*_*0*_ with the slope *b*. Using these results, for any dataset with sample size *N* and a desired p-value, the corresponding threshold can be obtained where *a* and *b* are fitted from the data. This threshold is used to remove statistically weak edges. Since each cytokine is explicitly an output we do not employ any further analysis such as DPI [18] to identify and remove indirect connections.

Using the network thus obtained, a predictive model can be developed as described in Appendix A.

### Application to phosphoprotein-cytokine signaling network

We employ this information theoretic approach to reconstruct the phosphoprotein-cytokine network in RAW 264.7 macrophages. To achieve this goal, the first step is the creation of the MI matrix (MIM) of interactions for each Toll and non-Toll data set separately and then finding a proper threshold for each network.

Macrophages play key roles in both innate and adaptive immunity, regulating the immune responses and the development of acute and chronic inflammations by producing a wide array of powerful chemical substances and regulatory factors such as cytokines [39]. Cytokines are a group of proteins and act as mediators between cells. Cytokines locate and interact with the target immune cells by binding to their receptor [40,41]. The release of immune-regulatory cytokines is regulated by a complex signaling network [34,42]. Multiple stimuli generate different signals and these signals generate different cytokine responses. Clear delineation of these signaling pathways is a prerequisite for understanding the causes of cytokine release.

#### Description of the data

In order to determine the signaling components involved in the cytokine release, we used the AfCS data on the phosphoproteins and cytokines under Toll and non-Toll conditions in RAW 264.7 macrophages. The phosphoprotein/cytokine data set consists of 22 phosphoproteins (inputs) and 7 cytokines (outputs). The cytokines (outputs) include: Tumor Necrosis Factor alpha (TNFα), Interleukin-1α (IL-1α), Interleukin-6 (IL-6), Interleukin-10 (IL-10), Granulocyte Macrophage Colony Stimulating Factor (GM-CSF), Regulated on Activation, Normal T Expressed and Secreted (RANTES) and Macrophage Inflammatory Protein- 1alpha (MIP-1α). The phosphoproteins (inputs) include: Signal Transducers and Activator of Transcription (STAT) 1α (STAT1α), STAT1β, STAT3, STAT5, Ribosomal Protein S6 (Rps6), Ribosomal S6 kinase (RSK), Glycogen Synthase Kinase (GSK) 3A (GSK3A), GSK3B, Extracellular-signal Regulated Kinases (ERK) 1 (ERK1), ERK2, cyclic Adenosine Monophosphate (cAMP), c-Jun N-terminal Kinases (JNK) long (JNK lg), JNK short (JNK sh), AKT, p40 Phagocyte Oxidase (p40Phox), Ezrin [Ezr]/Radixin [Rdx](Ezr/Rdx), Membrane-organizing Extension Spike Protein (Moesin or MSN), P38, Sma and Mad related proteins 2 (SMAD2), Nuclear Factor Kappa-light-chain-enhancer of activated B cells p65 (NF-κB p65), Protein Kinase C Delta (PKCD) and Protein kinase C μ2 (PKCμ2).

Both the input data and output data are time-averaged since the time-scales of the input data are in minutes (measurements taken at 1, 3 and 10 minutes) whereas that of the output (cytokines) data are in hours (measurements taken at 2, 3 and 4 hrs). Phosphoproteins were measured using western blots (AfCS protocols #PP00000177 and #PP00000181) and cytokines were measured using multiplex suspension arrays (AfCS protocols #PP00000209 and #PP00000223 [36]). More details about the experiments can be found on the AfCS website [36] and the procedure for pre-processing the data is explained by Pradervand et al. [34]. In short, phosphoprotein data corresponded to *log*(fold-change with respect to basal level) and cytokine data corresponded to *log*(treatment – control + 1).

The dataset used consists of Toll and non-Toll data. RAW cells were stimulated with a panel of 22 ligands, in single and double ligand combinations. The Toll data sets refers to the collection of data in which one of the ligands activates Toll-like receptors (TLRs) and results in major inflammation [34]. These TLR-ligands include lipopolysaccharide (LPS), Resiquimod (R-848), PAM2CSK4 (PAM 2) and PAM3CSK4 (PAM 3). The non-Toll data refers to the collection in which the ligands do not activate one or more of the TLRs. Due to the substantial extent of response when TLRs are activated by TLR-ligands (relative to other ligands), the important effect of other ligands gets masked if one of the ligands is a TLR-ligand. Hence, in order to identify the specific connections in the networks mediating information flow during stimulation by other ligands, the data was separated in Toll and non-Toll sets. After removal of rows with missing values across all inputs and outputs, Toll and non-Toll data each consisted of 78 rows or observations (78 × 22 input data matrix and 78 × 7 output data matrix), which were used to estimate MI in each case.

A reduced model of each set was obtained by applying the principles of information theory described above. Combining these two models, we obtained the network model based on the entire data set. The resulting network provides a parsimonious phosphoprotein-cytokine model, in which the number of signaling components involved in cytokine release is minimized considerably. This model not only successfully captures most of the known signaling components involved in cytokine release, but also predicts new signaling components involved in release of cytokines.

Finally, while not the main objective of this work, we also developed predictive models (albeit linear models since log-transformation removed some of the nonlinearity) using the significant inputs (MI > threshold). The procedure to develop the linear models is presented in Appendix A. We used the Toll dataset for developing the linear models. With the intent to validate the predictive linear models, the data set was partitioned in training and test sets. Since different sets of input variables are significant for different outputs, after eliminating the rows with missing values, the effective number of observations for each output was different, which ranged 89–115 for the training set and 33–39 for the test set; about 3:1 ratio for the number of training vs. test samples.

## Results

**Figure 1:**
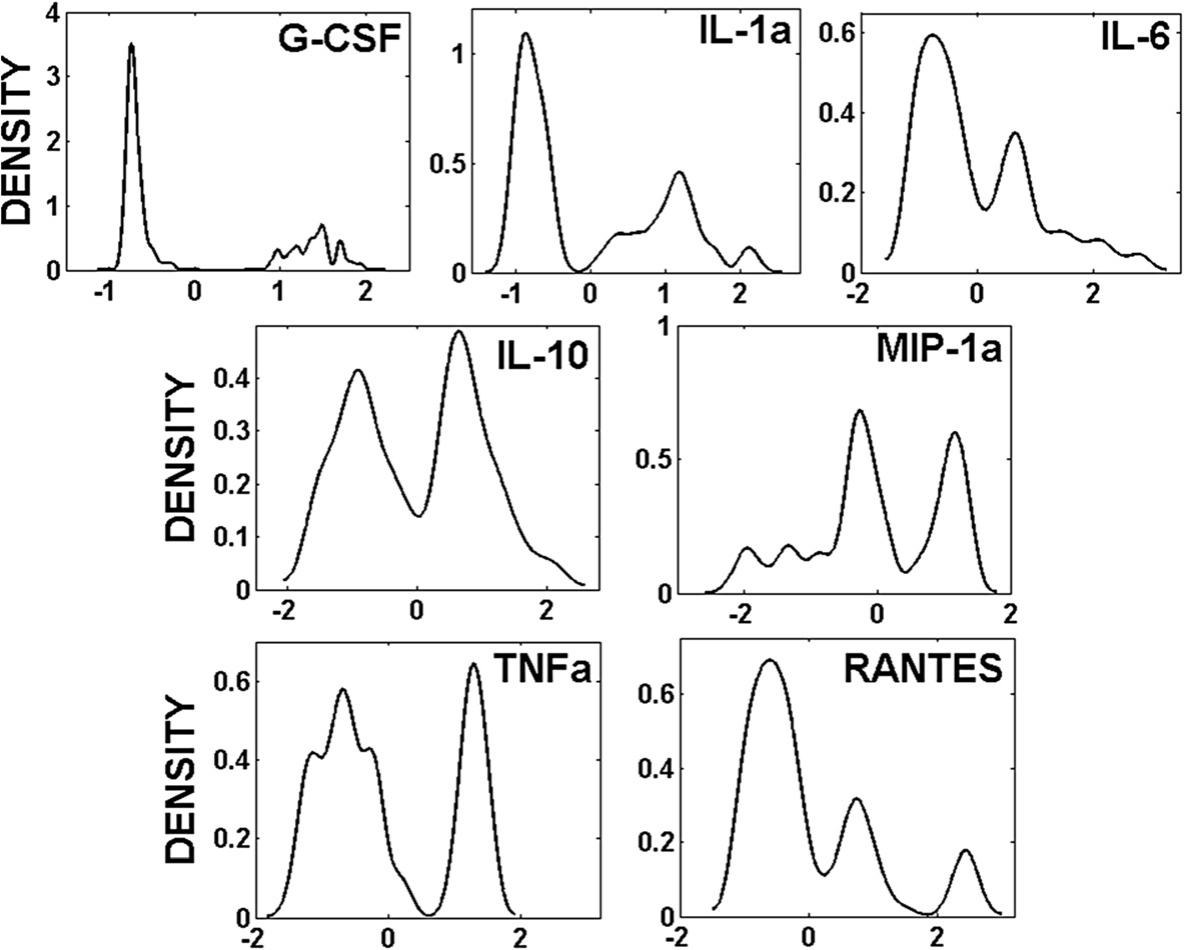
**Kernel density estimations (y-axis) of seven released cytokines (x-axis) in RAW264.7 macrophage cells upon stimulation with ligands, using kernel bandwidth *h *** **= 0.14 (Toll data).**

The proper kernel bandwidth has been estimated by applying the above-mentioned cross-validation approach (equation (7)). For Toll data set, the selected bandwidth (*h*) is 0.14 and for non-Toll data set, *h* is 0.17. Figure 1 shows the probability density functions of seven released cytokines, as inferred by the KDE in equation (3) computed through the MATLAB function *ksdensity*[43] using the estimated value of *h*. All of the estimated densities are highly non-Gaussian. In this figure, x-axis shows the measured values of cytokines after being normalized and the y-axis demonstrates their densities by applying KDE.

**Figure 2:**
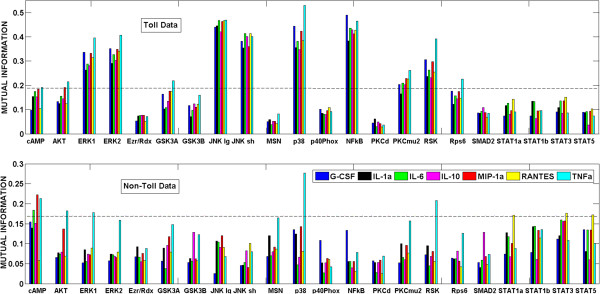
**Mutual information of all phosphoprotein-cytokine pairs from Toll (the upper bar plot) and non-Toll (the lower bar plot) datasets.** Thresholds (*I*_0_ = 0.19 for Toll data and *I*_0_ = 0.17 for non-Toll data) for p-value = 0.005 are shown by dashed lines.

Using these kernel density estimators, we used equation (3) to compute the MI coefficients of all protein-cytokine connections for the Toll and non-Toll datasets. Figure 2 shows these coefficients as a bar-graph, with the corresponding thresholds shown by the dashed lines for a p-value = 0.005 (*I*_0_ = 0.19 for Toll data and *I*_0_ = 0.17 for non-Toll data). The MI coefficients below these thresholds are considered to be statistically insignificant and discarded without any significant impact. It can be inferred from Figure 2 (both panels) that increase (decrease) in the desired p-value (and hence decrease (increase) in the MI threshold) will result in inclusion (exclusion) of some connections. For example, for non-Toll data, a small increase in MI threshold will make STAT1α and STAT5 insignificant for RANTES; connections to other cytokines will be unaffected. For the Toll data, since the MI values for the pairs cAMP – TNFα and AKT – MIP1α are close to the threshold, a small increase in the threshold will render these connections insignificant. Conversely, a small decrease in MI threshold will make the cAMP – MIP1α connection significant and hence be included in the network. Similar observations were made with the PCR approach as well [34]. Overall, with changing threshold, the network topology changes in a robust manner where just one or two edges appear or disappear.

**Figure 3:**
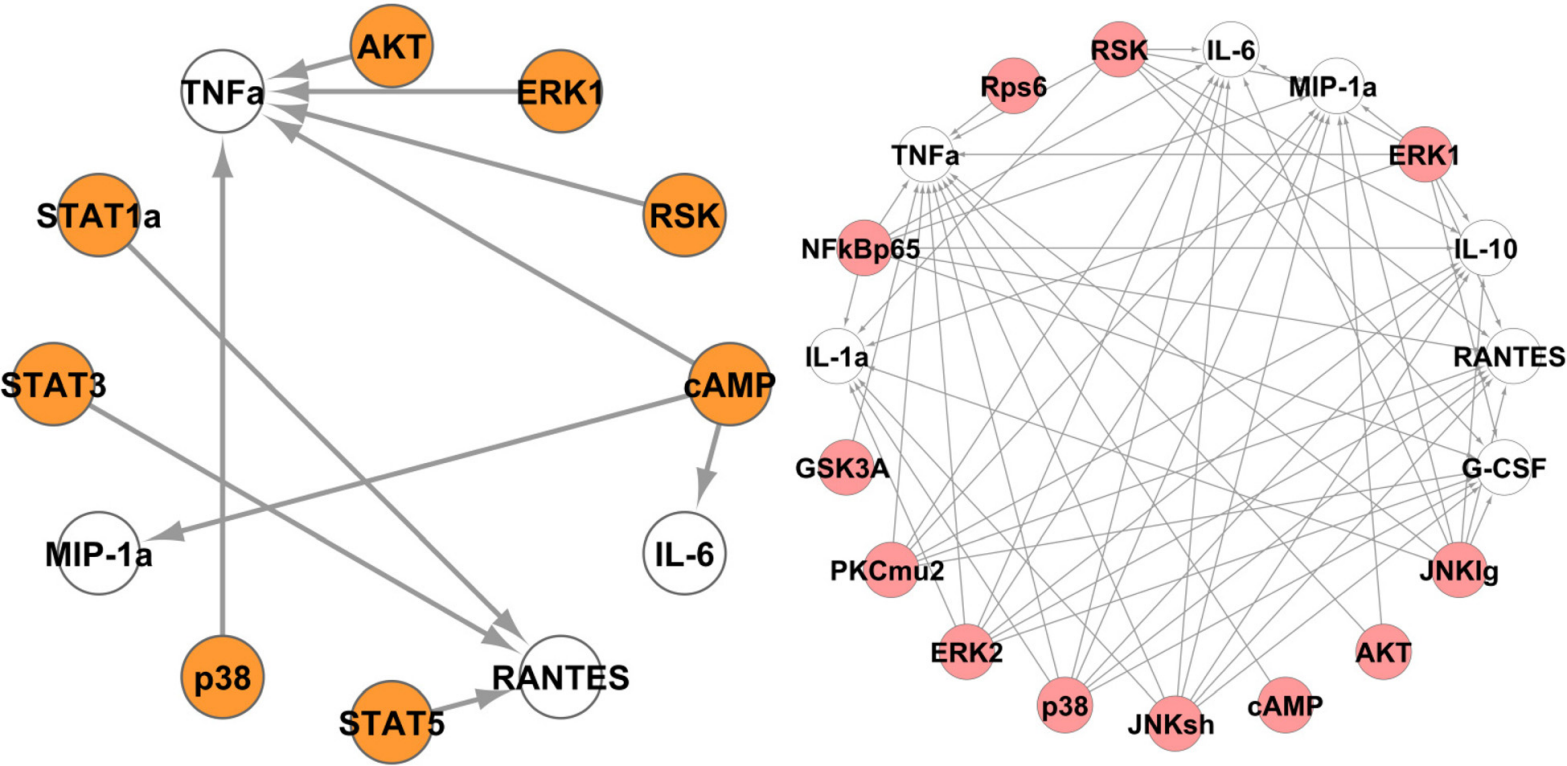
**Reconstructed networks of signaling phosphoproteins-cytokines obtained from the non-Toll (left panel with orange nodes for the phosphoproteins) and Toll (right panel with pink nodes for the phosphoproteins) data.** White circles indicate cytokine outputs. *h* = 0.14 and *I*_0_ = 0.19 for Toll data and *h* = 0.17 and *I*_0_ = 0.17 for non-Toll data.

**Figure 4:**
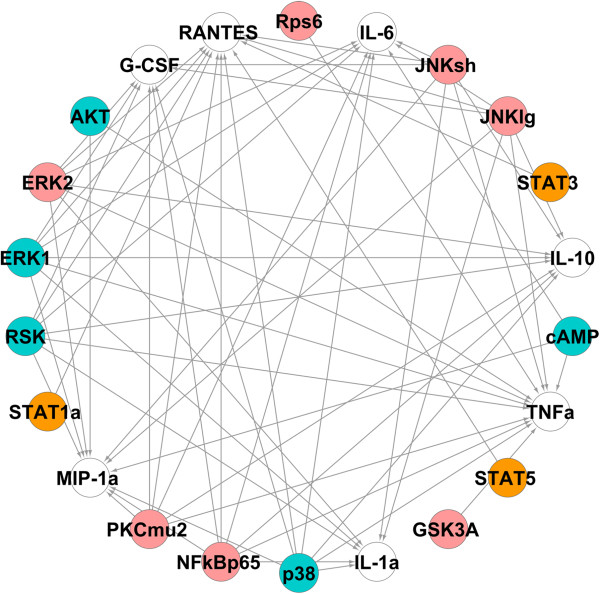
**The reconstructed phosphoprotein-cytokine network obtained by combining networks from non-Toll dataset (orange nodes) and Toll dataset (pink nodes).** Blue nodes are phosphoproteins involved in both datasets and white nodes represent the cytokines (outputs).

Figure 3 shows the reconstructed networks obtained from the non-Toll (left panel and orange nodes) and Toll (right panel and pink nodes) data for 22 signaling phosphoproteins and seven cytokines. These two networks are combined to yield the network of the entire system, which is shown in Figure 4. Blue nodes in Figure 4 show phosphoproteins involved in both datasets. This network captures most of the known signaling components involved in cytokine release and confirms the potentially important novel signaling components that have been suggested recently by other methods, such as PCR [34]. Our approach also identifies new signaling components involved in the release of cytokines, including Ribosomal S6 kinase on TNFα.

**Figure 5:**
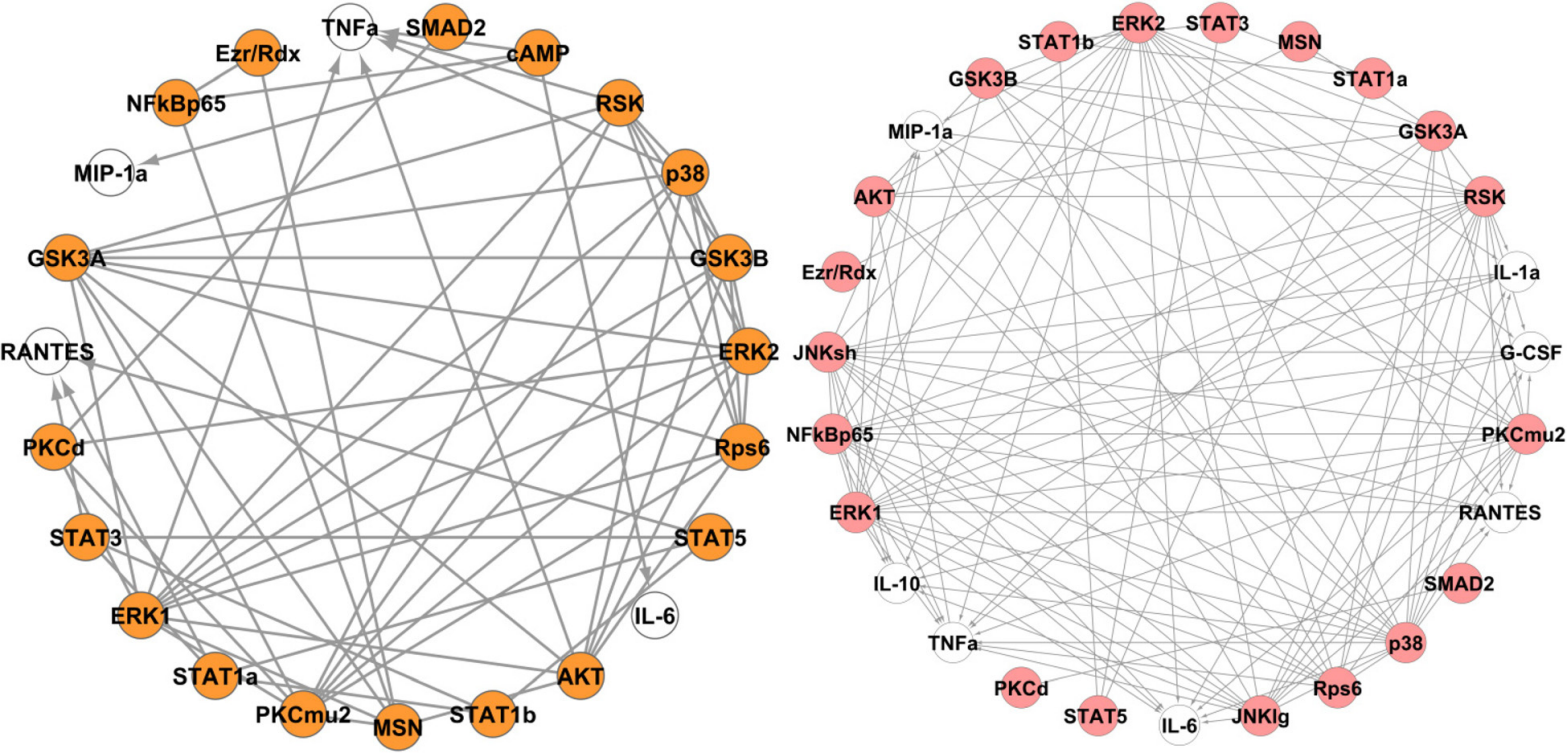
**Reconstructed networks of phosphoprotein-phosphoprotein/phosphoprotein-cytokine interactions obtained from the non-Toll (left panel and orange nodes) and Toll (right panel and pink nodes) data.** The white nodes represent the cytokines (*h* = 0.14 and *I*_0_ = 0.20 for Toll data and *h* = 0.17 and *I*_0_ = 0.17 for non-Toll data).

**Figure 6:**
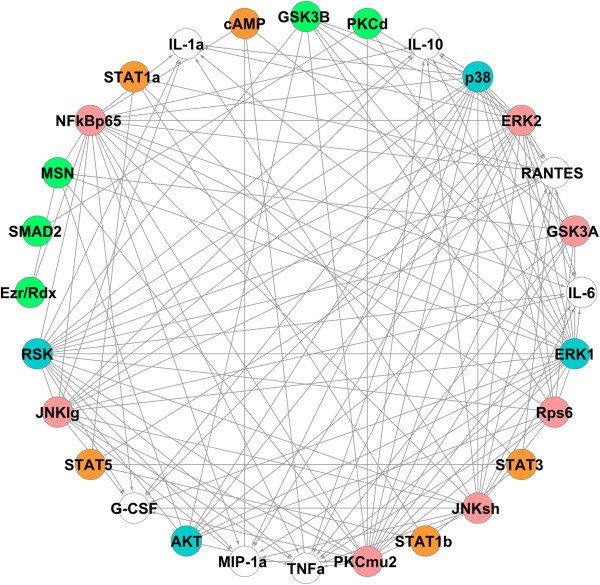
**The reconstructed phosphoprotein-phosphoprotein/cytokine network from combining networks from non-Toll dataset (orange) and Toll dataset (pink).** The white nodes represent the cytokines, blue nodes are involved in cytokine regulation from both datasets and green nodes are not directly involved in cytokine regulation.

**Figure 7:**
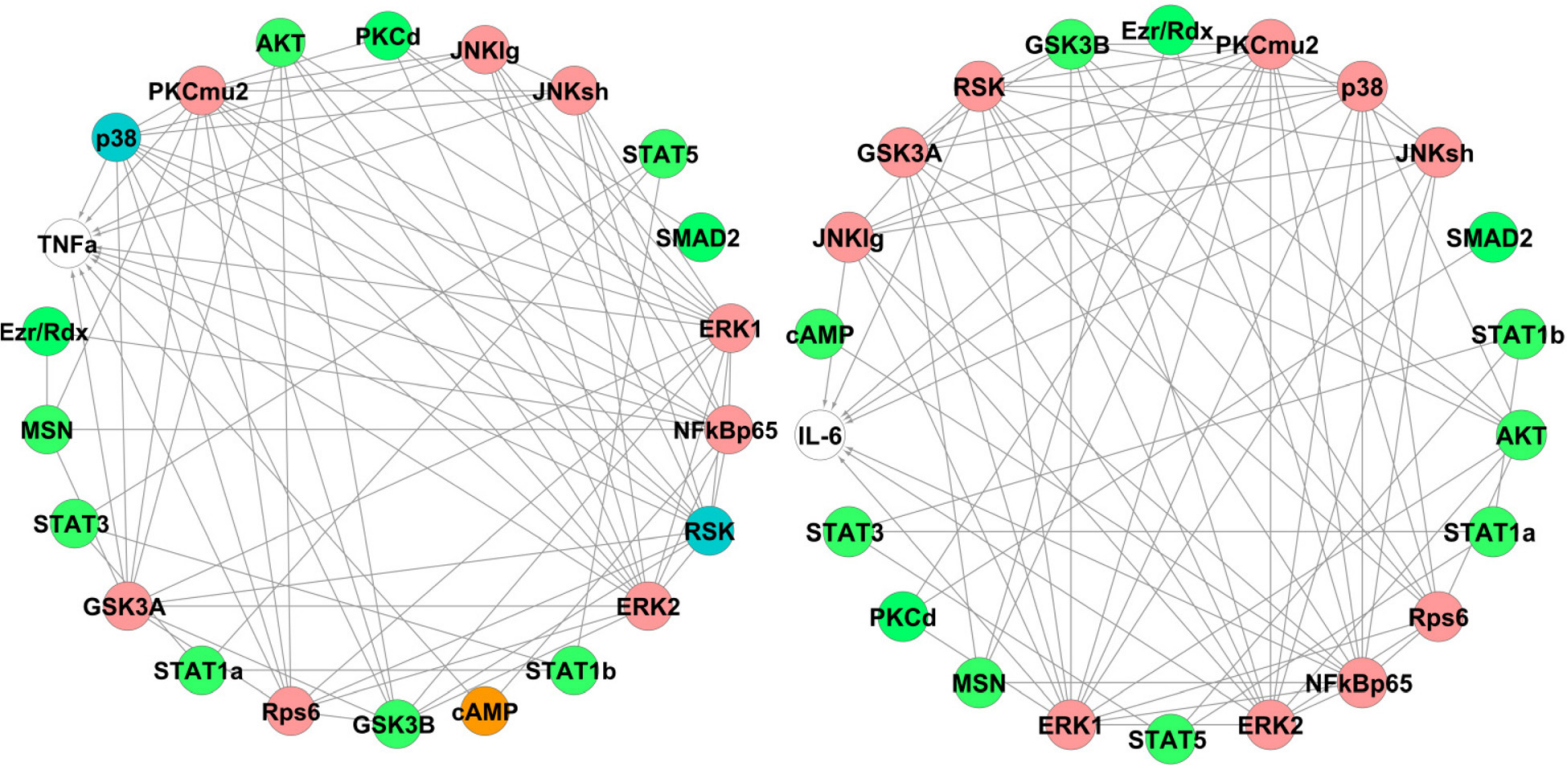
**Node-by-node reconstructed networks for TNFα (left panel) and IL-6 (right panel) after combining non-Toll dataset (orange nodes) and Toll dataset (pink nodes).** Blue nodes are involved in cytokine regulation from both datasets and green nodes are not directly involved in cytokine regulation.

Since phosphoproteins may also have regulatory impacts on other phosphoproteins, the above mentioned process is applied again to capture all the significant phosphoprotein-phosphoprotein and phosphoprotein-cytokine connections in one network. The mutual information matrix of all interactions is built again and the proper kernel bandwidth and threshold is selected (*h* = 0.14 and *I*_0_ = 0.20 for Toll data and *h* = 0.17 and *I*_0_ = 0.17 for non-Toll data). Figure 5 shows the reconstructed networks obtained from the non-Toll (left panel and orange nodes) and Toll (right panel and pink nodes) data and Figure 6 is the final network obtained by combining the two networks in Figure 5 containing significant phosphoprotein-phosphoprotein and phosphoprotein-cytokine connections in the entire system.To demonstrate the robustness of our results, this network is built again by capturing the networks of each cytokine individually and combining the seven reconstructed networks. Figure 7 shows the networks obtained from node-by-node analysis for TNFα (left panel) and IL-6 (right panel). In comparison with the network of Figure 6, such a network doesn’t capture the regulatory effect of PKCμ2 on G-CSF for Toll-data and cAMP on IL-6 and AKT on TNFα from non-Toll data. As the lower panel in Figure 2 shows, the mutual information of these interactions are very close to the selected threshold. All other connections present in Figure 6 are also included in such a network.

**Figure 8:**
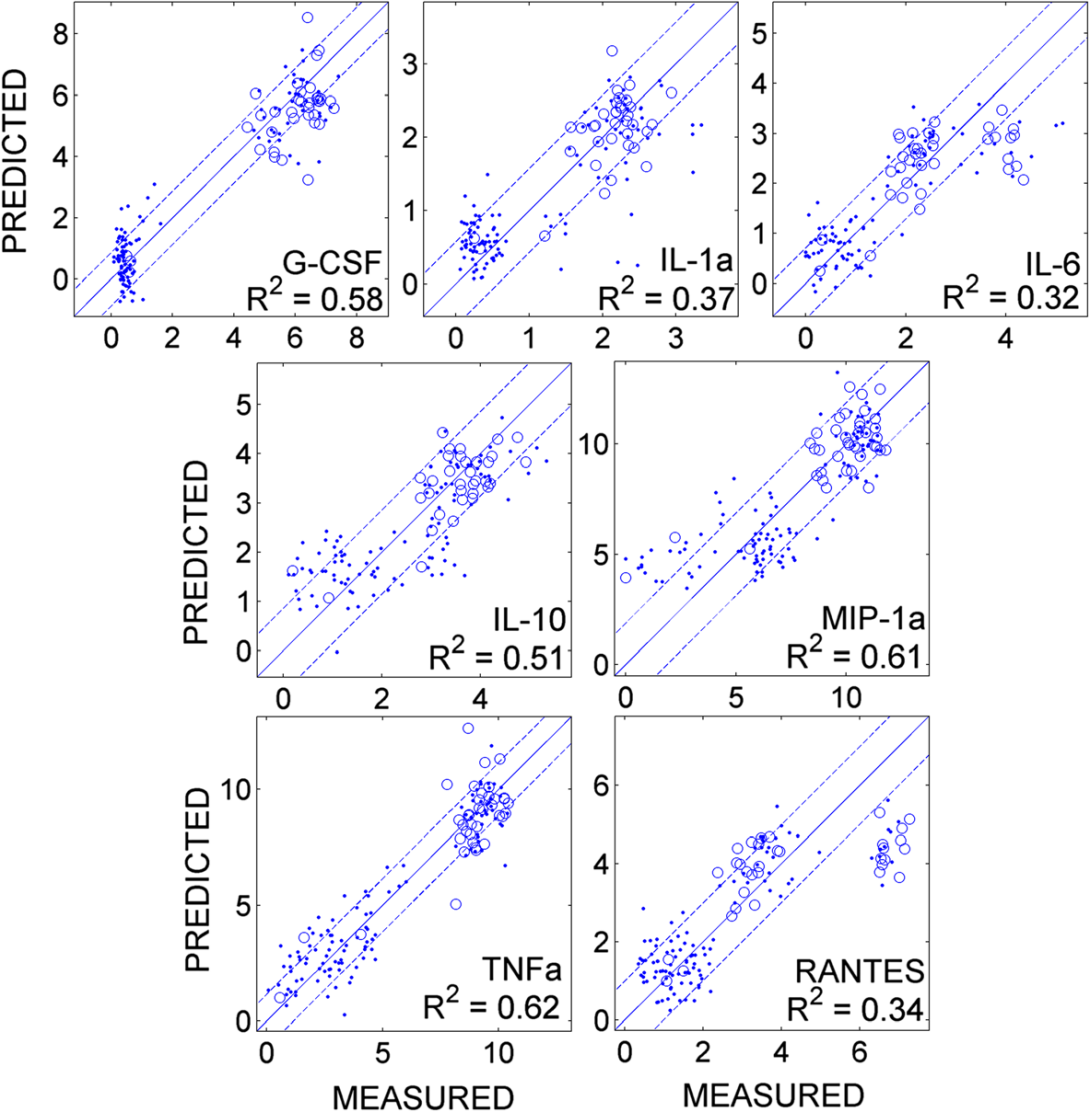
Predicted (y-axis) vs. measured (x-axis) values of training (dots) and test (open circles) data for the seven cytokines.

The scatter-plot in Figure 8 illustrates the predictive power of the linear models made from the reconstructed network from the Toll data (Figure 3, right panel) for training (dots) and test (open circles) datasets on cytokine release (see Methods). Most of the training and test data points are inside within two root-mean-squared errors of the training data (Appendix A). To provide a measure of the predictive quality of these linear models, we also computed the coefficient of determination *R*^*2*^ for each cytokine as described in Appendix A. The *R*^*2*^ values range from 0.32 to 0.62. TNFα and MIP-1α yield the best fit (*R*^*2*^ > 0.6) and IL-6 and RANTES yield the lowest coefficients of determination. Although the linear model derived based on the significant components identified through the information theoretic approach is in a good agreement with the predictive models obtained with other methods, such as PCR [34] and PLS [44], the low coefficient of determination in these models, even with log-transformation of the data, indicates the non-linear nature of the phosphoprotein-cytokine signaling networks.

## Discussion

The information theoretic approach accurately identifies the main signaling phosphoproteins involved in cytokine release (Figures 3 and 4). We analyzed both Toll and non-Toll ligand response datasets. Non-Toll data is required to identify the regulatory effects of STAT1α, STAT1β, STAT3, STAT5 and cAMP (Figure 3, left panel) and Toll-data provides information about PKCμ2, JNK lg, JNK sh and NF-κB P65 and ERK2 (Figure 3, right panel). ERK1, AKT, P38 and RSK are identified as significant in both datasets. We provide a comparison of the regulatory components necessary for cytokine release identified by the information theoretic approach and other computational methods such as PCR with statistical significance testing [34] and biochemical knowledge available in literature. The results of this comparison are summarized in Table 1.

**Table 1:** Significant components of phosphoprotein-cytokine signaling network

**Interactions**		**MI**	**PCR**	**Lit.**	**Comment**	**Interactions**		**MI**	**PCR**	**Lit.**	**Comment**
**G-CSF**	NF-κB	✓	✓	✓[44]		**IL-6**	RSK	✓	✘	✓[45]	- IL-6 has the lowest *R*^*2*^-value. This may be due to avoiding the linearity assumption in computing the mutual information.
(Pro-inflammatory)	JNK lg	✓	✓	✓[46]		(Anti-inflammatory and pro-inflammatory)	JNK lg	✓	✓	✓[47]	
	JNK sh	✓	✓	✓[46]			JNK sh	✓	✓	✓[47]	
	P38	✓	✓	✘			P38	✓	✘	✓[48]	
	PKCμ2	✓	✘	✓[49]			PKCμ2	✓	✘	✓[50]	
	ERK1	✓	✘	✓[51]			NF-κB	✓	✓	✓[52]	
	ERK2	✓	✘	✓[51]			ERK1	✓	✘	✓[45]	
	RSK	✓	✘	✓[53]			ERK2	✓	✘	✓[45]	
	**Finding:** Our model confirms the regulatory effect of P38, which has been suggested by the PCR minimal model [34].						cAMP	✓	✓	✓[54]	
**TNFα**	RSK	✓	✘	✘	- TNFα has the largest network. This study identifies the regulatory effect of 12 phosphoproteins on TNFα.	**IL-10**	JNK lg	✓	✓	✘	
(Pro-inflammatory)	AKT	✓	✓	✓[55]		(Anti-inflammatory)	P38	✓	✘	✓[56]	
	P38	✓	✓	✓[57]							
	RPS6	✓	✘	✓[58]							
	GSK3A	✓	✘	✓[59]	- TNFα yields the highest coefficient of determination *R*^*2*^*=* 0.62						
	GSK3B	✘	✘	✓[59]							
	PKCμ2	✓	✘	✓[60]			ERK1	✓	✘	✓[61]	
	cAMP	✓	✓	✓[62]			ERK2	✓	✘	✓[61]	
	NF-κB	✓	✓	✓[58]			JNK sh	✓	✓	✘	
	JNK lg	✓	✓	✓[63]			NF-κB	✓	✓	✓[64]	
	JNK sh	✓	✓	✓[63]			PKCμ2	✓	✘	✓[65]	
	ERK2	✓	✘	✓[66]			RSK	✓	✘	✓[67]	
	ERK1	✓	✘	✓[66]			**Finding:** Our study confirms the regulatory effects of JNK sh/ lg on IL-10.				
	**Finding:** Our study suggests the novel regulatory effect of RSK on TNFα.										
**MIP-1α**	P38	✓	✓	✓[57]	- This study doesn’t confirm the regulatory effects of STAT1α/β/3 on MIP-1α suggested by one study [68]. The PCR minimal model [34] identifies the impact of STAT1α on MIP-1a.	**RANTES**	STAT3	✓	✘	✓[69]	- Unlike the PCR minimal model [34], this study successfully captures the regulatory impacts of STAT1α/3/5 on RANTES.
(Anti-inflammatory)	NF-κB	✓	✓	✓[70]		(Anti-inflammatory)	STAT5	✓	✘	✓[71]	
	cAMP	✓	✓	✓[72]			STAT1α	✓	✓	✓[73]	
	RSK	✓	✘	✓[74]			NF-κB	✓	✓	✓[75]	
	JNK lg	✓	✓	✓[76]			P38	✓	✘	✓[77]	
	JNK sh	✓	✓	✓[76]			PKCμ2	✓	✘	✓[78]	
	AKT	✓	✘	✓[79]			JNK sh	✓	✓	✓[75]	
	ERK1	✓	✘	✓[74]			JNK lg	✓	✓	✓[75]	
	ERK2	✓	✘	✓[74]			RSK	✓	✘	✓[80]	
	STAT1α	✘	✓	✓[68]			ERK2	✓	✘	✓[80]	
	STAT1β	✘	✘	✓[68]			ERK1	✓	✘	✓[80]	
	STAT3	✘	✘	✓[68]		**IL-1α**	ERK2	✓	✘	✓[81]	
						(Pro-inflammatory)	ERK1	✓	✘	✓[81]	
							RSK	✓	✘	✓[82]	
							P38	✓	✘	✓[83]	
							JNK lg	✓	✓	✓[84]	
							JNK sh	✓	✓	✓[84]	
							NF-κB	✓	✓	✓[85]	

A comparison of phosphoprotein-cytokine regulatory connections identified by information theoretic approach (‘MI’), PCR (‘PCR’) and the literature knowledge (‘Lit.’). Identified interactions are shown by ✓ and cells marked by ✘ indicate missed interactions by a method.

Activated macrophages secrete cytokines [86]. Various pathways transmit the signals that initiate cytokine production [87,88]. Cytokines are classified based on their functions or their sources [86,89]. They can be grouped into anti-inflammatory and pro-inflammatory cytokines based on their functional role in inflammatory responses. Pro-inflammatory cytokines such as TNFα, IL-1α and GM-CSF induce both acute and chronic inflammatory responses. Anti-inflammatory cytokines, such as IL-10 limit the magnitude of inflammation and chemokines, such as MIP and RANTES are involved in chemotaxis of leukocytes.

### Pro-inflammatory cytokines

Granulocyte/macrophage Colony Stimulating Factor (G-CSF) regulates the production of neutrophil G granulocytes and stimulates the function of mature neutrophils [90]. We identify the phosphoproteins PKCμ2 [49], NF-κB p65 [44], JNK lg/sh [46], P38, RSK [53] and ERK1/2 [51] as the main regulators for the production and release of G-CSF. Tumor Necrosis Factor alpha (TNFα) is involved in normal host defense in both mediating inflammatory and immune responses [91]. Our study captures the largest network of regulatory components for TNFα which consists of twelve signaling phosphoproteins: RSK, AKT, RPS6, PKCμ2, GSK3A, cAMP, ERK1/2, JNK sh/lg, NF-κB p65 and P38. Some studies suggest the regulatory impact of STAT1α and STAT1β on TNFα [92]. Both our network and the network from PCR minimal model [34] missed these connections. Interleukin-1alpha (IL-1α) is produced by activated macrophages and is responsible for inflammation [93]. The information theoretic approach identifies cAMP, JNK lg/sh, ERK1/2, P38 and NF-κB p65 as the main regulators of production/release of IL-1α.

As Table 1 shows, this study identifies most of the signaling components of pro-inflammatory cytokines captured by other computational methods and strongly confirms the regulatory effect of P38 which has been proposed by the PCR minimal model [34]. Unlike the PCR minimal model [34], our approach successfully captures the regulatory effects of ERK1 and ERK2 on GCS-F [51] and TNFα [66]. It confirms the regulatory effect of GSK3A on TNFα [59] which have been suggested by studies. NF-κB, ERK, JNK (targets c-Jun [63]) and Sp1 (trans-activating transcription factor 1) are the transcriptional activators of TNFα [58,94]. In this light, our results show good agreement with other studies by capturing all signaling components identified by the PCR minimal model, in addition to predicting the known regulatory effects of ERK1/2, GSK3A (regulated by c-Jun which is affected by JNK) [44,59,94]. The information theoretic approach also identifies RSK, a substrate of ERK [95], as a potentially novel regulatory component involved in the release of TNFα.

P38 (from Toll data) has the strongest and ERK1 (from non-Toll data) has the weakest regulatory impact on TNFα. As Figure 8 shows, TNFα yields the best linear fit in terms of the coefficient of determination (*R*^*2*^ = 0.62), which is in good agreement with other models obtained by PCR [34,96] and PLS [44] methods. NF-κB p65 represents the highest statistical dependency while PKCμ2 has the lowest mutual information coefficient among the captured regulatory network components of GCS-F. JNK lg (from Toll data) shows the highest regulatory effect on IL-1α.

### Anti-inflammatory cytokines

Interleukin-10 (IL-10) is an anti-inflammatory cytokine that has important roles in immune regulation and inflammation [97]. Our approach shows the regulatory effects of PKCμ2 [65], P38 [56], RSK [67], ERK1/2 [61], NF-κB p65 [64] and JNK sh/lg, on IL-10. Macrophage Inflammatory Protein-1α (MIP-1α) belongs to the group of CC chemokines that regulate several inflammatory responses including trafficking and activation of leukocytes, as well as the fever response [98]. We capture the regulatory effects of cAMP [72], AKT [79], RSK, ERK1/2 [74], P38 [57], JNK sh/lg [76] and NF-κB p65 [70] on MIP-1α. One study suggests the regulatory effects of STAT1α/β and STAT3 on MIP-1α [68]. The PCR minimal model [34] only identifies STAT1α as a significant component of MIP-1α. Regulated on Activation, Normal T Expressed and Secreted (RANTES), is a CC chemokine and has a key role in recruiting leukocytes into inflammatory sites [99]. The information theoretic approach suggests that STAT3, STAT5, STAT1α, NF-κB p65, PKCμ2, P38 JNK lg/sh, ERK1/2 and RSK regulate RANTES and unlike the PCR minimal model [34], it is in good agreement with the cytokine literature.

As indicated in Table 1, the network identified by our study includes most of the known signaling components of anti-inflammatory cytokines described in the literature and unlike the PCR minimal model [34], captures the regulatory effects of NF-κB p65 and ERK1/2 on MIP-1α. Some studies suggest that the TLR ligand pathways that activate IL-10 are P38 dependent and NF-κB signaling pathway has no contribution on the activation of IL-10 [100,101]. However, our study and the PCR model [34] identify the regulatory effects of JNK lg/sh which are activated through NF-κB p65.

The information theoretic approach and PCR [34] models both yield low coefficient of determination for cytokines (*R*^*2*^ < 0.8) possibly due to their regulations by unmeasured pathways and/or a nonlinear relationship between the levels of cytokines and the phosphoproteins. In comparison to the PCR approach, information theoretic approach shows a better agreement with known regulatory components in the literature. The high variability of data (low coefficient of determination) might explain this by considering the fact that when noise or variability is high, the threshold used in the PCR approach is high so that it identifies a relatively lesser number of components as being significant. The non-linear nature of the biological processes might be an explanation for the failure of PCR to identify the regulatory effects of ERK1/2, cAMP and RSK on cytokines.

JNK lg (from Toll data) has the strongest effect and AKT (from non-Toll data) has the weakest effect on MIP-1α. Our network shows the highest mutual information (from non-Toll data) for NF-κB and IL-10. PKCμ2 has the weakest regulatory effect on IL-10. JNK lg has the strongest regulatory effect on RANTES and STAT3 shows the lowest statistical dependencies to it.

### Interleukin-6

Interleukin-6 (IL-6) is secreted by macrophages and T cells and acts as both a pro-inflammatory and anti-inflammatory cytokine [102]. Our model identifies the regulatory effects of phosphoproteins RSK, PKCμ2, ERK1/2, JNK sh/lg, P38, NF-κB and cAMP. The regulatory roles of cAMP [54] and P38 [48] which could not be captured by the PCR minimal model [34], are identified by the information theoretic approach. JNK lg (from Toll data) yields the strongest regulatory effect and cAMP (from non-Toll data) yields the weakest regulatory effect on IL-6.

Overall, our network model and quantitative predictions are in good agreement with other studies available in literature and captures most of the known regulatory components involved in cytokine release. Our model confirms the regulatory effect of P38 on G-CSF that has been suggested by the PCR minimal model several years ago [34] and captures one potentially novel regulatory effect of RSK on TNFα. The advantages of the information theoretic method has been demonstrated by comparing the accuracy of its parsimonious model to the models obtained by other computational methods such as PCR minimal models in predicting the regulatory components for cytokines with high variability and low coefficient of determination.

## Conclusions

Identifying the regulatory components for cytokines is critical for understanding the mechanisms that control their production and release in immune cells. In recent years, several computational methods have been applied to develop signaling networks, involved in cytokine release, which have led to an improved understanding of cytokine release in macrophages. In this work, we developed a parsimonious input–output model of regulatory phosphoprotein-cytokine network based on an information theoretic approach. Our model demonstrated the applicability of this approach to the data-driven reconstruction of biological networks. The data, which consisted of a systematic profiling of signaling responses in RAW 264.7 macrophage cells upon treatment with Toll- and non-Toll receptor ligands, was provided by the Alliance for Cellular Signaling (AfCS). Information theoretic approach as a non-parametric method identified the regulatory components (phosphoproteins) on which specific cytokines showed significant statistical dependence (measured in terms of mutual information). The reconstructed network also was able to capture the regulatory network of phosphoprotein interactions. We calculated mutual information of interactions by using kernel density estimator (KDE) and discarded weak connections using proper thresholds. Using such a parsimonious list of significant inputs, a predictive model was also developed for each of the cytokines which predicted a separate test data well. Most of the significant connections are validated against the known literature. Some novel connections, such as Ribosomal S6 kinase for Tumor Necrosis Factor alpha are also identified by the mutual information approach, which were not detected by the PCR approach. These novel regulatory components serve as testable hypotheses.

### Availability of supporting data

The data sets supporting the results of this article are available at the UCSD Signaling Gateway web site [http://www.signaling-gateway.org/data/Data.html].

## Appendices

### Appendix A - development of a predictive model

To develop a predictive model using the reconstructed network, we build the following linear model between the significant inputs (*X*) and a chosen output (*Y*):

(10) Y=X.b+ϵ

where, *ϵ* represents white noise. Generally, one deals with one output at a time because the set of significant inputs differs for different outputs. *X* is mean-centered and normalized by the standard deviation and *Y* is mean-centered. The coefficient matrix, *b*, is estimated by least square method [103] using “training dataset”:

(11) $$\hat{b}={\left(X^{T}.X\right)}^{-1}.\left(X^{T}.Y\right)$$

Once $\hat{b}$ is estimated, the model can be tested on a “test dataset”. The test dataset generally has the same probability distribution as training dataset. Thus, given the input test data *X*_test_ (normalized by using the mean and standard deviation parameters obtained for the training set), the output test data *Y*_test_ (offset by the mean of *Y*) is predicted as:

(12) $$Y_{\mathrm{test},\mathrm{pred}}=X_{\mathrm{test}}.\hat{b}$$

Two metrics used to measure the accuracy of the prediction are Root Mean Square Error (RSME) and coefficient of determination (*R*^*2*^) calculated as [104]:

(13) $$\mathrm{RMS}E_{\mathrm{test}}=\sqrt{\frac{1}{n}\sum_{i=1}^{n}(Y_{\mathrm{test},i}-Y_{\mathrm{test},i,\mathrm{pred}}){}^{2}}$$

(14) $$R^{2}=1-\frac{\sum_{i=1}^{n}{\left(Y_{\mathrm{test},i}-Y_{\mathrm{test},i,\mathrm{pred}}\right)}^{2}}{\sum_{i=1}^{n}{\left(Y_{\mathrm{test},i}-{\bar{Y}}_{\mathrm{test}}\right)}^{2}}$$

where, *n* is the number of data points. ${\bar{Y}}_{\mathrm{test}}$ is the mean value of the *n* data points for the chosen output. *R*^*2*^ is a good quantitative metric indicating the quality of prediction by the linear model.

## Competing interests

The authors declare that they have no competing interests.

## Authors’ contributions

Research design and supervision: SS, DMT, MRM. Algorithm: FF, MRM, SS, DMT. Computer program: FF, MRM. Writing and revision: FF, MRM, SS, DMT. FF and MRM contributed equally to this work. All authors read and approved the final manuscript.
